# Supplementary material for: Coronin-1 promotes directional cell rearrangement in Drosophila wing epithelium
Source: Cell Struct Funct. 2023 Nov 30;48(2):251–7. doi: 10.1247/csf.23049 (PMC11496784; doi:10.1247/csf.23049)
Supplement: Supplementary file 1 — Supplementary Materials [file csf_48_23049_1.pdf]

Supplementary Methods

Image collection

To prepare the *Drosophila* pupal wing samples for image collection, pupae at around 24 hour after puparium formation (h APF) were fixed to double-sided tape and the pupal case above the left wing was removed (Guirao *et al.*, 2015, Ikawa and Sugimura, 2018). The pupae were then placed on a small drop of water or Immersol W 2010 (444969-0000-000, Zeiss, Oberkochen, Germany) in a glass bottom dish with the left side facing downward. The fixed time-point and timelapse images of Coronin-1-EGFP were acquired using a confocal microscope (LSM900; Zeiss Oberkochen, Germany) equipped with a 63×/NA1.2 C-Apochromat water-immersion objective at room temperature. Images of phalloidin staining were acquired using a confocal microscope (LSM900; Zeiss Oberkochen, Germany) equipped with a 40×/NA1.3 C-Apochromat oil-immersion objective. Images for the analysis of cell rearrangement were acquired using an inverted confocal spinning disk microscope (Olympus IX83 combined with Yokogawa CSU-W1; Olympus, Tokyo, Japan; Yokogawa, Tokyo, Japan) equipped with an iXon3 888 EMCCD camera (Oxford Instruments, Abingdon-on-Thames, UK), an Olympus 60×/NA1.2 SplanApo water-immersion objective (Olympus, Tokyo, Japan) and a temperature control chamber (TOKAI HIT, Shizuoka, Japan), using IQ 2.9.1 (Oxford Instruments, Abingdon-on-Thames, UK) (Guirao *et al.*, 2015). Images for the analysis of hexagonal cell packing were acquired using an inverted confocal microscope (A1R; Nikon, Tokyo, Japan) equipped with a 60×/NA1.2 Plan Apochromat water-immersion objective at 25°C. After imaging, we confirmed that the pupae survived to at least the pharate stage.

Phalloidin staining

Pupae at appropriate ages were dissected, and wings were fixed at room temperature for 30 min in PBS containing 4% paraformaldehyde. After washing thrice with PBS containing 0.1% Triton X-100 (PBT), these preparations were blocked with PBT containing normal goat serum (005-000-121, Jackson ImmunoResearch, Pennsylvania, U.S.A) (PBTn) and then incubated overnight with Alexa Fluor 647 Phalloidin (1/500; A22287, Thermo Fisher, Massachusetts, U.S.A) diluted in PBTn. After washing with PBT three times, we mounted these preparations on a glass slide for observation.

Drosophila genetics

The flies used in the present study were *flr-GFP* (Flytrap #CA07499) (Buszczak *et al.*, 2007), *DE-cad-GFP* (Huang *et al.*, 2009), *sqhp-sqh-mKate2×3* (Pinheiro *et al.*, 2017), *ptc-Gal4*, *UAS-flr dsRNA* (VDRC #v108422), *UAS-coro dsRNA* (BDSC #40841), *coro-EGFP* (this study), UAS-*tsr* dsRNA (VDRC #v110599). Fly genotypes and culture conditions are summarized below. Since the efficacy of the *coro* RNAi line (BDSC #40841) was validated and shown to be stronger than another *coro* RNAi line (VDRC

#v109644), particularly in terms of the change in F-actin intensity (Xie *et al.*, 2021), we used the BDSC *coro* RNAi line throughout this study.

Genotype	Culture condition	Figure
coro-EGFP/sqh-mKate2×3	Crossed and observed at 25°C (24 h APF)	Fig. 1C, E, F, G and H
ptc-Gal4, *coro-EGFP/UAS-coro dsRNA*	Flies were crossed at 17°C. White pupae were picked up and observed at 25°C (24 h APF)	Fig. 1D
DE-cad-GFP	Flies were crossed at 17°C. White pupae were picked up and observed at 25°C (24 h APF)	Fig. 2B and C
ptc-Gal4, *DE-cad-GFP/DE-cad-GFP*, *UAS-coro dsRNA*	Flies were crossed at 17°C. White pupae were picked up and observed at 25°C (24 h APF)	Fig. 2B and D
ptc-Gal4, *sqh-mKate2×3/+*; *Flare-GFP/+*	Flies were crossed at 17°C. White pupae were picked up and observed at 25°C (24 h APF)	Fig. 3A
ptc-Gal4, *sqh-mKate2×3/UAS-coro dsRNA*; *Flare-GFP/+*	Flies were crossed at 17°C. White pupae were picked up and observed at 25°C (24 h APF)	Fig. 3B
coro-EGFP/sqh-mKate2×3	Crossed and observed at 25°C (24 h APF)	Fig. 3C
ptc-Gal4, *coro-EGFP/sqh-mKate2×3*, *UAS-flr dsRNA*	White pupae were picked up and observed at 17°C. (58 h APF at 17°C)	Fig. 3D
ptc-Gal4, *coro-EGFP/tg80^{ts}*, *UAS-tsr dsRNA*	Flies were crossed at 20°C. White pupae were picked up and fixed at 29°C (21 h APF)	Fig. 3E–H
ptc-Gal4, *coro-EGFP/tg80^{ts}*, *UAS-tsr dsRNA*	Flies were crossed at 20°C. White pupae were picked up and observed at 29°C (21 h APF)	Supplementary Fig. 1

Supplementary table 1 Fly genotypes and culture conditions.

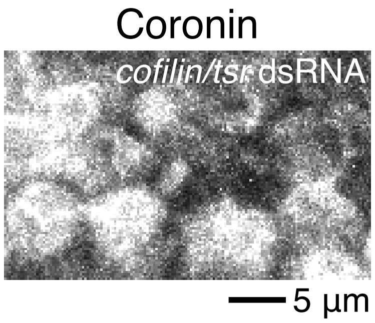

Supplementary Figure 1

Images of Coronin-1-EGFP in the C region of the wing expressing *cofilin/tsr* dsRNA using *ptc*-Gal4 at 21 h APF at 29°C, which corresponds to 24 h APF at 25°C.

Scale bar: 5 µm

Supplementary References

Buszczak, M., Paterno, S., Lighthouse, D., Bachman, J., Planck, J., Owen, S., Skora, A.D., Nystul, T.G., Ohlstein, B., Allen, A., Wilhelm, J.E., Murphy, T.D., Levis, R.W., Matunis, E., Srivali, N., Hoskins, R.A. and Spradling, A.C. 2007. The Carnegie protein Trap Library: A versatile tool for *Drosophila* developmental studies. *Genetics*, **175**: 1505–1531.

Guirao, B., Rigaud, S.U., Bosveld, F., Bailles, A., López-Gay, J., Ishihara, S., Sugimura, K., Graner, F. and Bellaïche, Y. 2015. Unified quantitative characterization of epithelial tissue development. *Elife*, **4**: e08519

Huang, J., Zhou, W., Dong, W., Watson, A.M. and Hong, Y. 2009. Directed, efficient, and versatile modifications of the *Drosophila* genome by genomic engineering. *Proc. Natl. Acad. Sci.*, **106**: 8284–8289.

Pinheiro, D., Hannezo, E., Herszterg, S., Bosveld, F., Gaugue, I., Balakireva, M., Wang, Z., Cristo, I., Rigaud, S.U., Markova, O. and Bellaïche, Y. 2017. Transmission of cytokinesis forces via E-cadherin dilution and actomyosin flows. *Nature*, **545**: 103–107.

Xie, Y., Budhathoki, R., Blankenship, T.J. 2021. Combinatorial deployment of F-actin regulators to build complex 3D actin structures *in vivo*. *Elife*, **10**: e63046
